# Heparin and Carboxymethylchitosan Metal Nanoparticles: An Evaluation of Their Cytotoxicity

**DOI:** 10.1155/2013/314091

**Published:** 2013-02-18

**Authors:** Adriana Bava, Francesca Cappellini, Elisa Pedretti, Federica Rossi, Enrico Caruso, Elena Vismara, Maurizio Chiriva-Internati, Giovanni Bernardini, Rosalba Gornati

**Affiliations:** ^1^Dipartimento di Biotecnologie e Scienze della Vita, Università dell'Insubria, 21100 Varese, Italy; ^2^Dipartimento di Scienze Teoriche ed Applicate, Università dell'Insubria, 21100 Varese, Italy; ^3^Dipartimento di Chimica, Materiali e Ingegneria Chimica “G. Natta,” Politecnico di Milano, 20131 Milano, Italy; ^4^Interuniversity Center “The Protein Factory,” Politecnico di Milano, ICRM-CNR Milano and Università dell'Insubria, 20131 Milano, Italy; ^5^Division of Oncology and Hematology, Texas Tech University Health Sciences Center, Lubbock, TX 79409, USA

## Abstract

In the search for noninvasive diagnostic techniques and new therapies, “nanosystems”, which are capable of binding and targeting bioactive molecules, are becoming increasingly important. In this context, biocompatible coatings are gaining interest, not only for their biological effects but also because they are considered capable to mask nanoparticle toxicity. In this work, we have compared the toxicity of nanoparticles coated with heparin and carboxymethylchitosan in the SKOV-3 cell line. Our results indicate that heparin and carboxymethylchitosan coatings do not guarantee the decrease of nanoparticle intrinsic toxicity which is often envisaged. Nonetheless, these coatings provide the opportunity for further functionalization with a variety of biomolecules for their use in theranostics.

## 1. Introduction 

Nanomedicine, the application of nanotechnology in healthcare, offers numerous and promising possibilities to significantly improve medical diagnosis and therapy. New sensitive diagnostic devices, in fact, will permit very early personal risk assessment, and the abatement of costs for the disease treatment is a must for healthcare. Due to its high potential, nanomedicine holds the promise to greatly improve the efficacy of pharmaceutical therapy, reduce side effects, and make drug administration more convenient [1].

In this context, nanoparticles (NPs), particularly magnetic nanoparticles (MNPs), coated with biodegradable polymers, are attracting widespread attention for targeted therapy and imaging. These coatings can stabilize the NP systems also in hydrophilic fluids, minimize opsonization by the mononuclear phagocytic system, and prolong blood circulation [2–7]. Furthermore, this surface layer can be functionalized with a variety of biological moieties for tumor-specific targeting [8–10].

Among the biological molecules used for NP coating, chitosan, particularly carboxymethylchitosan (CMCS), and heparin appear very interesting also because they are considered capable to mask NP toxicity [11, 12]. We should not, in fact, oversee the toxicity of cobalt and nickel oxide NPs [13–16] nor their potential effect on the environment [17]. Even though heparin is predominantly used as anticoagulant, its ability to interact with proteins makes it very attractive. NPs coated with heparin (NP@heparin) are extensively studied because of their several biomedical applications ranging from tissue engineering to biosensors passing for its use in cancer therapy [3]. As well as heparin, also chitosan NPs have demonstrated anticancer activity *in vitro *as well as *in vivo* even though the mechanisms remain to be elucidated [18].

In this paper, we have reported cytotoxicity and uptake of some transition metal oxide NPs (Co_3_O_4_, Fe_3_O_4_, and NiO) coated with heparin and of Fe_3_O_4 _NPs coated with CMCS (Fe_3_O_4_@CMCS) in SKOV-3 cell. Transition metal NPs are especially used to enhance surface electrochemical reactivity to further improve the performance of lithium-ion batteries [19] as well as in catalysis [20, 21]. Nevertheless, the therapeutic use of transition metal conjugates was already known in the sixteenth century because of their different oxidation states and ability to interact with negatively charged molecules forming chelation complexes [22]. 

The results here reported indicate that heparin and CMCS alone did not show any cytotoxicity effect at the concentration used in the experiments. Unfortunately, they did not seem to be able to drastically reduce NP toxicity.

## 2. Materials and Methods

### 2.1. Chemicals

Iron oxide (Fe_3_O_4_), cobalt oxide (Co_3_O_4_) and nickel oxide (NiO) NPs (<50 nm particle size), chitosan powder (75% degree of acetylation), monochloroacetic acid, 1-ethyl-3-(3-dimethylaminopropyl)carbodiimide hydrochloride (EDC·HCl), and N-hydroxysuccinimide (NHS) were purchased from Sigma-Aldrich, Milan, Italy. Heparin, in the form of sodium salt, was kindly provided by LDO Company, Trino Vercellese, Italy. CellTiter-Glo Luminescent Cell Viability Assay was purchased from Promega, Milan, Italy. Isopropanol was purchased from J.T.Baker, Milan, Italy. All other reagents, analytical or cell culture grade, were purchased from Sigma-Aldrich, Milan, Italy. The Milli-Q ultrapure water was used. 

### 2.2. Nanoparticles Characterization

The particle size distribution was studied by transmission electron microscopy (TEM) using a 90 keV JEOL-1010 electron microscope (Tokyo, Japan). TEM samples were prepared by placing 10 *μ*L of a dilute suspension of Fe_3_O_4_ nanoparticles in ethanol on a carbon-coated copper grid and allowing the solvent to evaporate at room temperature. The average particle size (*D*_TEM_) and distribution were evaluated by measuring the largest internal dimension of 100 particles.

### 2.3. Synthesis of Carboxymethylchitosan (CMCS)

**Figure 1:**
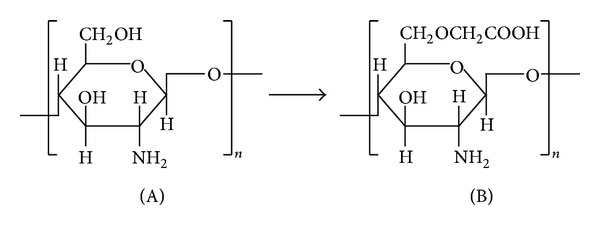
Chemical structure of chitosan (A) and carboxymethylchitosan (B).

CMCS was prepared as reported by Zhu et al. [23]. The chemical structures of chitosan and CMCS are reported in Figure 1.

### 2.4. Coating of Metal Nanoparticles

#### 2.4.1. NP@heparin

A suspension of NPs (Co_3_O_4_ or Fe_3_O_4_ or NiO) in distilled water (100 mg/5 mL) obtained by ultrasonication for 5 min (Sonica 5300MH-Soltec) was transferred into a solution of heparin, (1.045 g/25 mL, pH 7 adjusted with 0.01 N NaOH). The mixture was stirred overnight (130 rpm, 25°C, Julabo SW22). Co_3_O_4_@heparin and NiO@heparin were separated by centrifugation (1 h, 6300 ×g, Hettich Zentrifugen-Rotina 35 F), while Fe_3_O_4_@heparin was separated by a neodymium magnet (NdFeB Nickel plated, magnetization N45). NP@heparin were collected in diethyl ether, recovered after solvent elimination and dried at 50°C for 1 h.

#### 2.4.2. Fe_3_O_4_@CMCS Electrostatic Bound

A suspension of 100 mg of Fe_3_O_4_NPs in 5 mL water was prepared by ultrasonic bath for 10 min. Separately, 100 mg of CMCS was dissolved in 20 mL of water using a magnetic stirrer until complete dissolution, then added to the Fe_3_O_4_ NPs dispersion and mixed by ultrasonic bath at 0°C for 1 h. After reaction, the Fe_3_O_4_@CMCS was separated from unbound CMCS by a neodymium magnet, washed several times with water, and centrifuged at 15000 ×g, 20 min. The pellet was resuspended in ethyl alcohol then, after anhydrification, Fe_3_O_4_@CMCS was dried overnight at 50°C. 

#### 2.4.3. Fe_3_O_4_@CMCS-Covalent Bound

The covalent immobilization of CMCS on Fe_3_O_4_NPs was conducted following Liang and Zhang method [24] with some modifications. Briefly, 75 mg of Fe_3_O_4_NPs were added to 4 mL of sodium phosphate buffer (200 mM, pH 5) containing 25 mg of EDC*·*HCl and 20 mg of NHS; the mixture was left in an ultrasonic bath for 30 min. The Fe_3_O_4_NPs activated were separated from excess of reagents by magnetic decantation, then resuspended in 3 mL of 200 mM sodium phosphate buffer (pH 7) by sonication for 10 min. 1 mL of CMCS solution (25 mg/mL in 200 mM sodium phosphate buffer, pH 7) was added to the suspension of the NPs and the reaction mixture was sonicated for 3 h. Finally, the Fe_3_O_4_@CMCS was recovered by magnet, washed with water, and dried overnight at 50°C.

#### 2.4.4. Determination of Unbound Fe

 5 mg of Fe_3_O_4_NPs, or Fe_3_O_4_@heparin, or Fe_3_O_4_@CMCS electrostatically bound or Fe_3_O_4_@CMCS covalently bound were resuspended in 5 mL of H_2_O, sonicated for 20 min, and left at 37°C for 72 h. Afterwards, NP systems were separated from the supernatant by a neodymium magnet, centrifuged twice at 15000 ×g for 15 min at 4°C, then ultracentrifuged at 300000 ×g for 2 h at 4°C. After centrifugation, supernatants were filtered using a 0.22 *μ*m pore size membrane. The amount of Fe (II), eventually released in solution, was determined by complexometric analysis with the *o*-phenanthroline [25]. The Fe (II), in the presence of *o*-phenanthroline, form the stable red-orange complex [(C_12_H_18_N_2_)_3_Fe]^2+^. The intensity of the color does not vary in the range of pH between 3 and 9. The maximum absorption wavelength occurs at 510 nm. The possible Fe (III) is reduced to Fe (II) by treatment with hydroxylamine hydrochloride.

### 2.5. FT-IR Spectra Analysis

Characterization of the samples was performed using the solid phase Fourier transform infrared spectroscopy (FT-IR). Spectra were obtained using a Nicolet, Avatar 360. Samples were mixed with infrared grade KBr in a proportion of 2 : 100 (w/w).

### 2.6. Cell Culture

SKOV-3 cell line was maintained as adherent cells in RPMI 1640 medium, at 37°C in a humidified 5% CO_2_ atmosphere. Medium was supplemented with 10% fetal bovine serum and 2 mM L-glutamine. Cells were passaged as needed using 0.5% trypsin EDTA. 

### 2.7. Cell Viability

Cell viability was determined measuring ATP content by the CellTiter-Glo Assay according to the manufacturer's instructions. In details, 200 *μ*L of cell suspension (containing 2 × 10^4^, 1 × 10^4^, 5 × 10^3^, 25 × 10^2^ cells depending of the exposure time) were seeded into 96-well assay plates and cultivated for 24 h at 37°C in 5% CO_2_ to equilibrate and become attached prior to the treatment. Then, cells were exposed to 100 *μ*L of increasing concentrations of heparin, NP@heparin, CMCS, Fe_3_O_4_@CMCS electrostatic, and Fe_3_O_4_@CMCS covalent for 0.5, 1, 2, 24, 48, and 72 h. After the treatment, plates were equilibrated for 30 min at room temperature and then 100 *μ*L of CellTiter-Glo reagent was added to each well. Plates were shaken for 2 min and left at room temperature for 10 min prior recording luminescent signals using the Infinite F200 plate reader (Tecan Group, Switzerland). Cell viability, expressed as ATP content and normalized against control values, was recorded. All the experiments were performed in triplicate.

### 2.8. Cellular Uptake

10^4^ cells were seeded on a coverslip (12 mm Ø) into 12-well assay plate and cultivated for 24 h at 37°C in 5% CO_2_ to equilibrate and become attached before treatment. Cells were then incubated for 4 or 24 h with 25, 50, and 100 *μ*g/mL Fe_3_O_4_@heparin, Fe_3_O_4_@CMCS electrostatic, and Fe_3_O_4_@CMCS covalent and visualized by Prussian blue staining for iron detection. For this microscopic technique, the cells were fixed in ice-cold ethanol for 5 min, stained with an equal volume of 2% hydrochloric acid and 2% potassium ferrocyanide trihydrate for 15 min, and counterstained with 0.5% neutral red for 3 min. The preparations were then washed with distilled water and dried by increasing concentrations of ethanol, than mounted in DePeX (Serva, Germany). Observations were performed by a Zeiss Axiophot microscope under bright light illumination and photographs were acquired by a Zeiss AxioCam ERc5s camera. 

Furthermore, for TEM studies, 10^6^ cells, seeded in a 10 cm Petri dish, are exposed to 40 *μ*g/mL of NP@heparin, Fe_3_O_4_@CMCS electrostatic, and Fe_3_O_4_@CMCS covalent, for 30 min or 3 h. Then cells were harvested, fixed in 2% glutaraldehyde in 0.1 M sodium cacodylate buffer (pH 7.2) for 10 min on ice and for 30 min at room temperature, washed in the same buffer, and postfixed in dark for 1 h with 1% osmium tetroxide in 0.1 M sodium-cacodylate buffer (pH 7.2) at room temperature. After dehydration standard steps with a series ethyl alcohol, samples were embedded in an Epon-Araldite 812 1 : 1 mixture. Thin sections (90 nm), obtained with a ReichertUltracut S Ultratome (Leica, Nussloch, Germany), were stained with uranyl acetate and lead citrate according to the standard methods and observed with a Jeol 1010 electron microscope (Jeol, Tokyo, Japan) operated at 90 keV.

### 2.9. Statistical Analysis

Cell viability values were expressed as mean ± standard error (SE). Analysis of variance (two-way ANOVA), for balanced mixed-effect experiments (uncoated NPs, coated NPs, and exposure times), was performed using KaleidaGraph 4.0 (Synergy Software). Statistical significant differences were fixed at *P* ≤ 0.05 (*), *P* ≤ 0.01 (**), and *P* ≤ 0.005 (***).

## 3. Results

### 3.1. Nanoparticles Characterization

To confirm the characteristics reported on the product label by Sigma-Aldrich, we have measured Fe_3_O_4_NPs diameter. *D*_TEM_ was 25.08 nm ± SD 4.09. The amount of Fe released from Fe_3_O_4_NPs, or Fe_3_O_4_@heparin, or Fe_3_O_4_@CMCS-electrostatic bound or Fe_3_O_4_@CMCS-covalent bound, in our experimental conditions, was under the limit of detection of the method (0.02 ppm).

### 3.2. FT-IR Spectra Analysis

**Figure 2:**
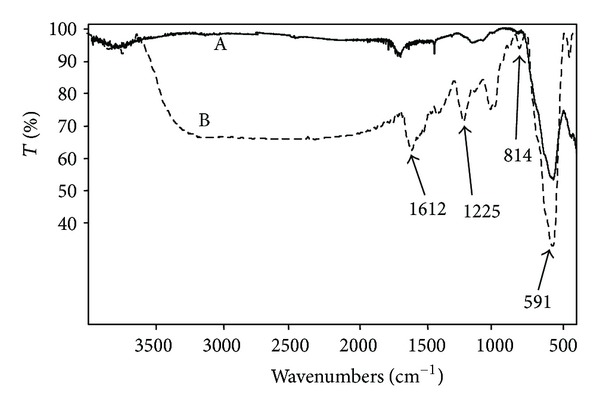
FT-IR spectra of Fe_3_O_4 _NPs (A) and Fe_3_O_4_@heparin (B). The peaks at 814 cm^−1^, between 1000 and 1400 cm^−1^ and those at 1225, and 1612 cm^−1^ are indicative of the heparin coating.

In Figure 2, we have reported, as example, the FT-IR spectra of Fe_3_O_4_NPs (A) and Fe_3_O_4_@heparin (B). Spectrum (B) shows, at 591 cm^−1^, the characteristic peak of Fe–O stretch, while, between 1000 and 1400 cm^−1^, peaks associated to C–O and C–C bonds due to the presence of heparin are present. Other peaks at 814, 1225, and 1612 cm^−1^ can be assigned to the stretching of –C–O–S, –S=O, and –COO^−^ of the sulphates and carboxylate groups. Lambda shifts toward lower values compared to heparin alone are probably ascribable to the interaction with iron oxide.

**Figure 3:**
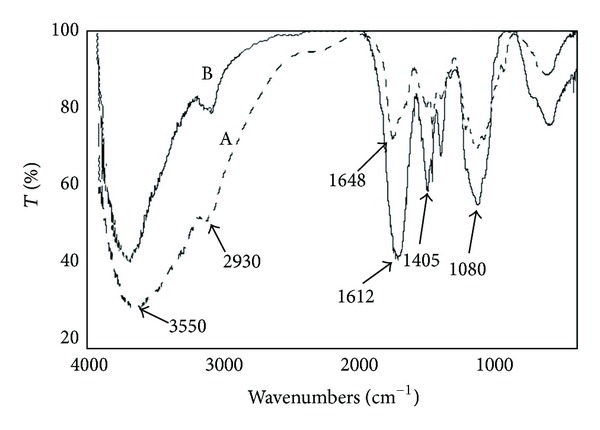
FT-IR spectra chitosan (A) and CMCS (B). Chemical modification of chitosan is confirmed by the presence of the intense peak at 1612 cm^−1^, attributed to the overlapping of the signals of NH bending and –COO^−^.

FT-IR spectra of chitosan (A) and CMCS (B) are shown in Figure 3. Spectrum A shows the basic characteristic peaks of chitosan: 3550 cm^−1^ (O–H stretch), 2930 cm^−1^ (C–H stretch), 1648 cm^−1^ (NH bending), 1405 cm^−1^ (O–H bending), and 1080 cm^−1^ (C–O stretch). In addition to the peaks at 3550, 2930, 1405, and 1080 cm^−1^, CMCS spectrum (B) reports an expanded and intense peak at 1612 cm^−1^ probably due to the overlapping of the signals of NH bending (1648 cm^−1^) and COO^−^ (1598 cm^−1^) [23]. 

**Figure 4:**
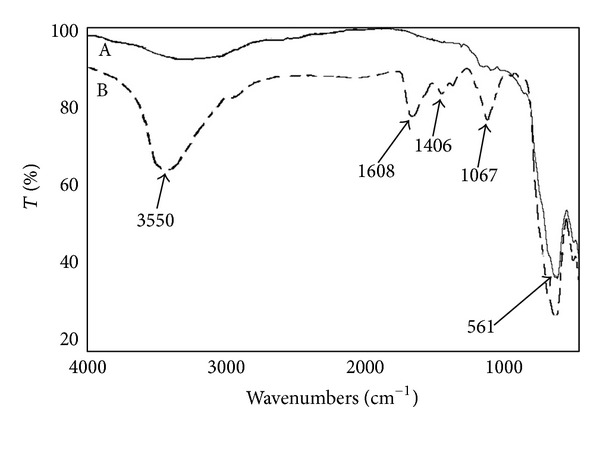
FT-IR spectra Fe_3_O_4 _NPs (A) and Fe_3_O_4_@CMCS (B). The peaks at 1067, 1406, 1608, and 3550 cm^−1^ indicate the presence of CMCS on the Fe_3_O_4_NPs surface.

Fe_3_O_4_NPs (A) and Fe_3_O_4_@CMCS (B) spectra are shown in Figure 4. In spectrum A, the peak at 561 cm^−1^ is that characteristic of Fe–O stretch. Spectrum B, beside to the peak at 561 cm^−1^, reports the absorbance of CMCS molecule, in particular 1067 cm^−1^ (C–O stretch), 1406 cm^−1^ (O–H bend), 1608 cm^−1^ (overlapping of the peaks of NH_2_, COOH, and COO^−^), and 3550 cm^−1^ (O–H stretch). No significant differences were observed between spectra of Fe_3_O_4_@CMCS electrostatically and covalently bound.

### 3.3. Cell Viability after NP@heparin Treatment

**Figure 5:**
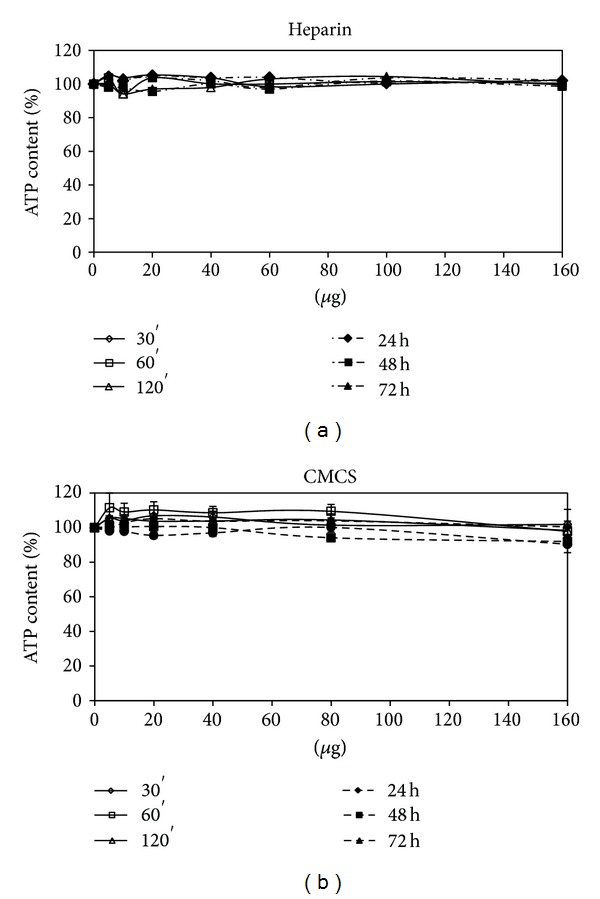
Percentage of ATP content, normalized to control, in SKOV-3 exposed to heparin (a) and carboxymethylchitosan (b) for different times.

**Figure 6:**
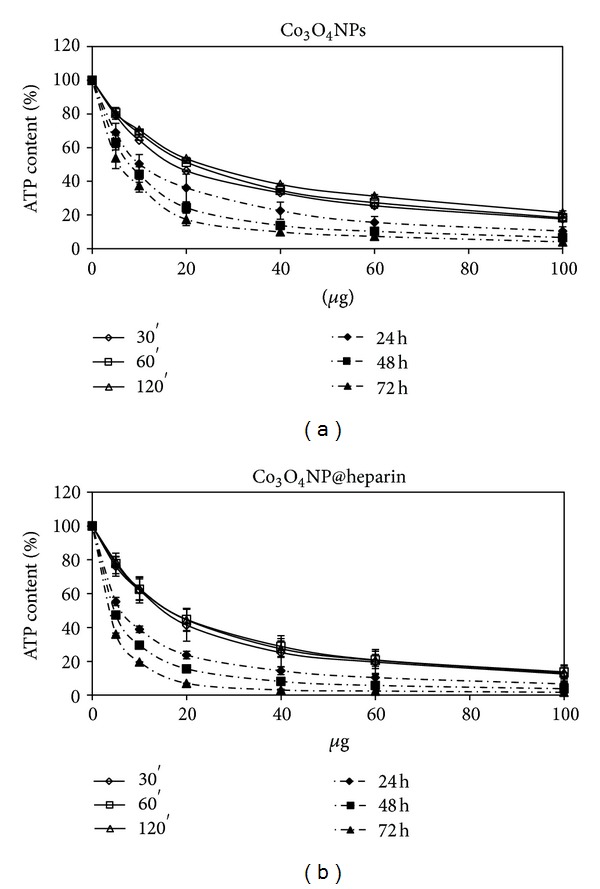
Percentage of ATP content, normalized to control, in SKOV-3 exposed to Co_3_O_4 _NPs (a) and Co_3_O_4_@heparin (b) for different times.

**Figure 7:**
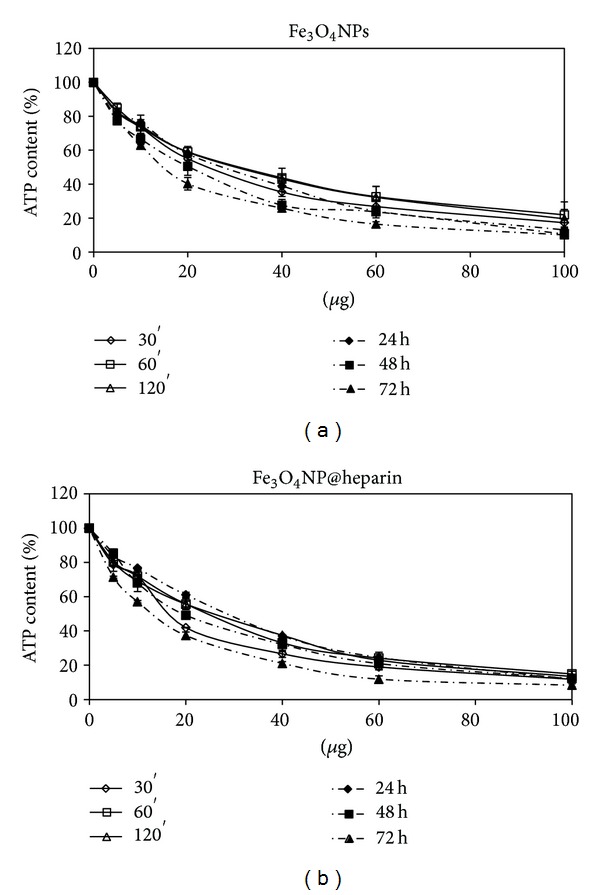
Percentage of ATP content, normalized to control, in SKOV-3 exposed to Fe_3_O_4 _NPs (a) and Fe_3_O_4_@heparin (b) for different times.

**Figure 8:**
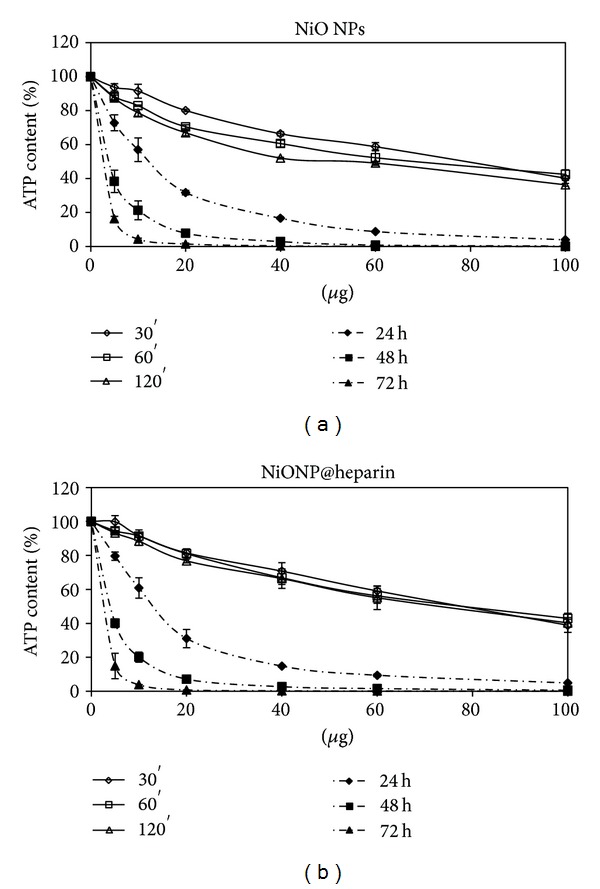
Percentage of ATP content, normalized to control, in SKOV-3 exposed to NiO NPs (a) and NiO@heparin (b) for different times.

As reported in Figure 5(a), heparin alone was not toxic in the examined concentration range. A dose- and time-dependent reduction in cell viability was observed for all the examined NP systems, although Fe_3_O_4_NPs appear less toxic than Co_3_O_4_ NPs which is less toxic than NiO NPs, see Figures 6(a), 7(a), and 8(a).

Regarding the comparison between uncoated and coated NPs, our data indicate that the coating did not decrease the NPs toxicity. As demonstrated in Figure 6, Co_3_O_4_NPs were less toxic than Co_3_O_4_@heparin for all the examined concentrations and time of treatment. The differences were less indicative for Fe_3_O_4_NPs and NiO NPs (Figures 7 and 8). For further details, see Supplementary Material Tables 1, 2, and 3 available online at http://dx.doi.org/10.1155/2013/314091.

### 3.4. Cell Viability after Fe_3_O_4_@CMCS Treatment

**Figure 9:**
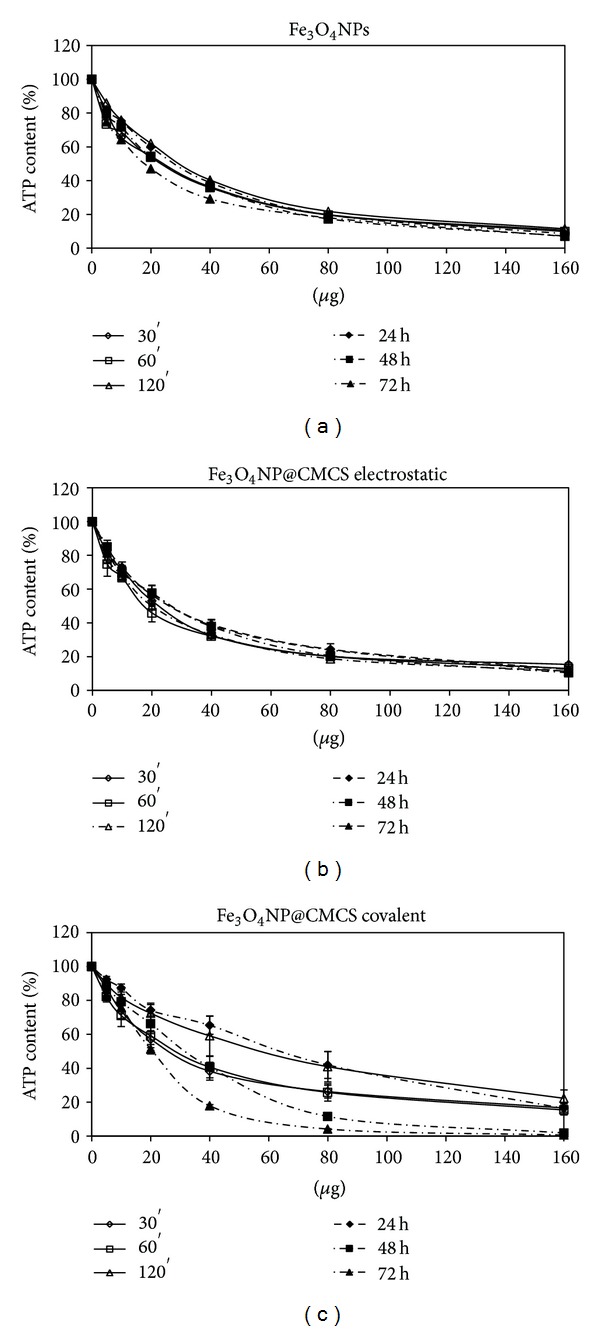
Percentage of ATP content, normalized to control, in SKOV-3 exposed to Fe_3_O_4 _NPs (a), Fe_3_O_4_@CMCS electrostatic (b), and Fe_3_O_4_@CMCS covalent (c) for different times.

The ATP content of SKOV-3 treated with Fe_3_O_4_NPs, Fe_3_O_4_@CMCS-electrostatic, and Fe_3_O_4_@CMCS covalent are displayed in Figure 9. The percentage of CMCS bound to NPs was less than 4% of the total weight; therefore, it was reasonable to compare the amount of coated and uncoated Fe_3_O_4_NPs neglecting the weight of CMCS bound. As previously reported (Figure 5(b)), CMCS itself did not show cytotoxicity at the tested concentrations. On the contrary, Fe_3_O_4_@CMCS covalent, and electrostatic, caused a dose-dependent reduction of ATP (Figures 9(a), 9(b) and 9(c)) more pronounced compared to the bare Fe_3_O_4_NPs. For further details see Supplementary Material Table 4. 

### 3.5. Cellular Uptake

**Figure 10:**
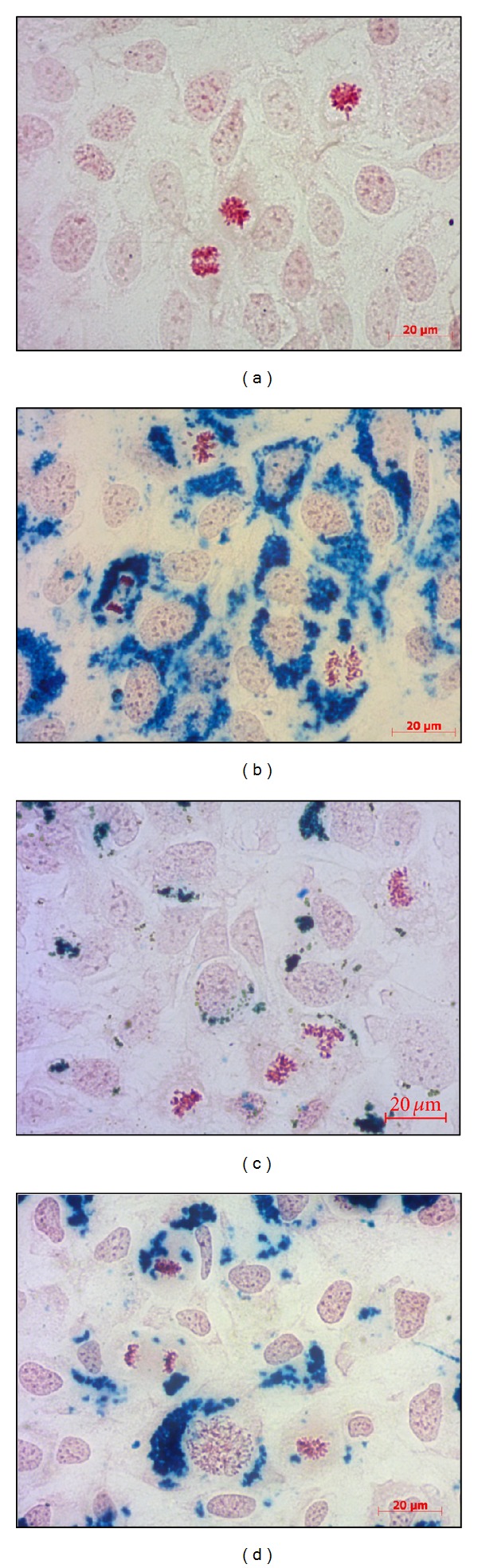
Uptake of SKOV-3 cells after 24 h of incubation with 25 mg/mL of anionic NPs. (a) Control cells; (b) Fe_3_O_4_@CMCS covalent; (c) Fe_3_O_4_@CMCS electrostatic; (d) Fe_3_O_4_@heparin. As shown in (b, c, and d) internalized NPs did not interfere with mitosis process. Magnification: 40x.

Figures 10(a)–10(d) show the uptake of coated Fe_3_O_4_NPs by using the classical Prussian blue method. The cytoplasm is full of NPs around the nucleus but never inside. Fe_3_O_4_@heparin (Figure 10(d)) are more internalized compared to Fe_3_O_4_@CMCS covalent (Figure 10(b)) and electrostatic (Figure 10(c)). Apparently, no differences are observed between the two chitosan systems. Coated Fe_3_O_4 _NPs are readily incorporated into the cells already after 4 h; therefore, it is not possible to assert a time and dose dependence. In addition, for all the NP systems, it is observed that internalized NP did not interfere with mitosis process (Figures 10(b)–10(d)).

**Figure 11:**
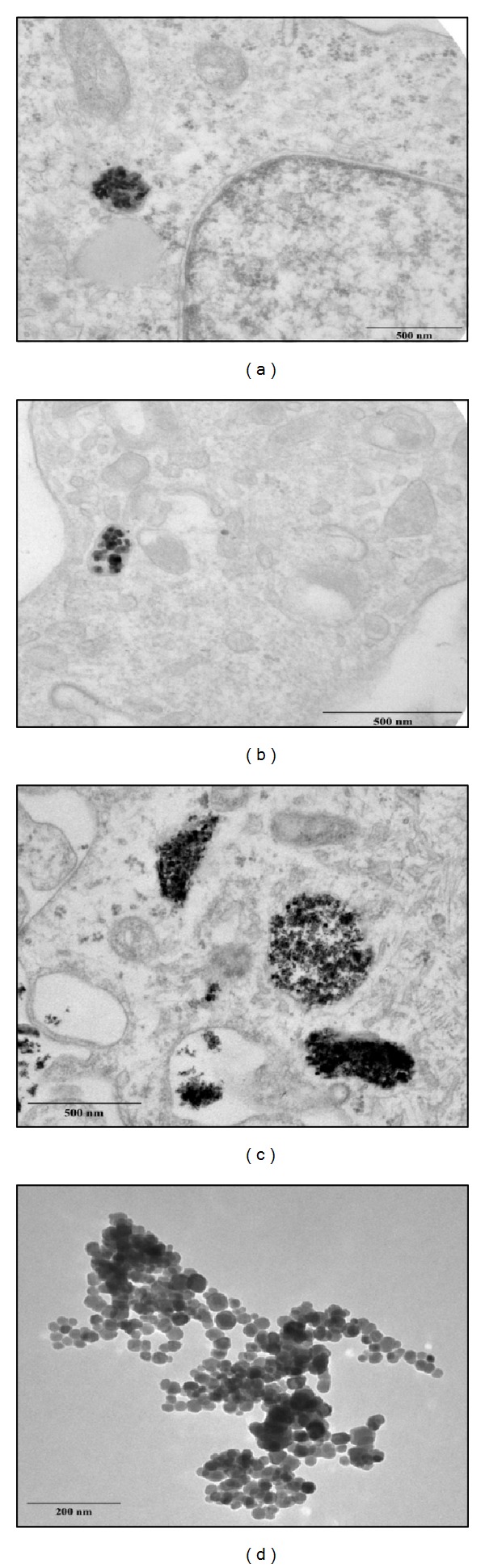
TEM pictures of SKOV-3 cells exposed to Co_3_O_4_@heparin (a), Fe_3_O_4_@heparin (b), and NiO@heparin (c) for 30 min. (d) A picture of Fe_3_O_4_@heparin deposited on a formvar carbon coated grid.

**Figure 12:**
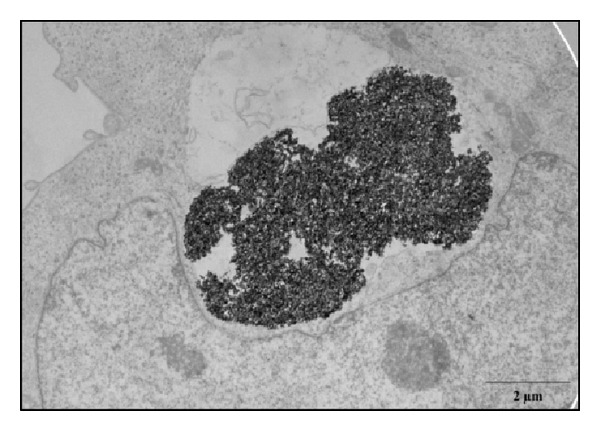
TEM picture showing a large agglomerate of Co_3_O_4_@heparin which modifies the shape of a SKOV-3 nucleus. Cells were fixed after 30 min of exposure.

TEM images (Figure 11) confirmed that NP@heparin are readily internalized; in fact, already after 30 min of incubation NPs appeared inside the cells. Once entered most of the NPs remained in the cytoplasm, free or inside vesicles (Figures 11(a)–11(c)). As already highlighted by optical microscope, besides being rapid, internalization of the nanoparticles was aspecific. In these pictures, NP@heparin are identified as high electron density objects since NPs maintained the morphology observed in cell-free environment (Figure 11(d)). Worth to note is that, also after 3 h of exposure, no NP@heparin was observed in the nuclei even though the massive internalization of NPs can modify nucleus shape (Figure 12). 

**Figure 13:**
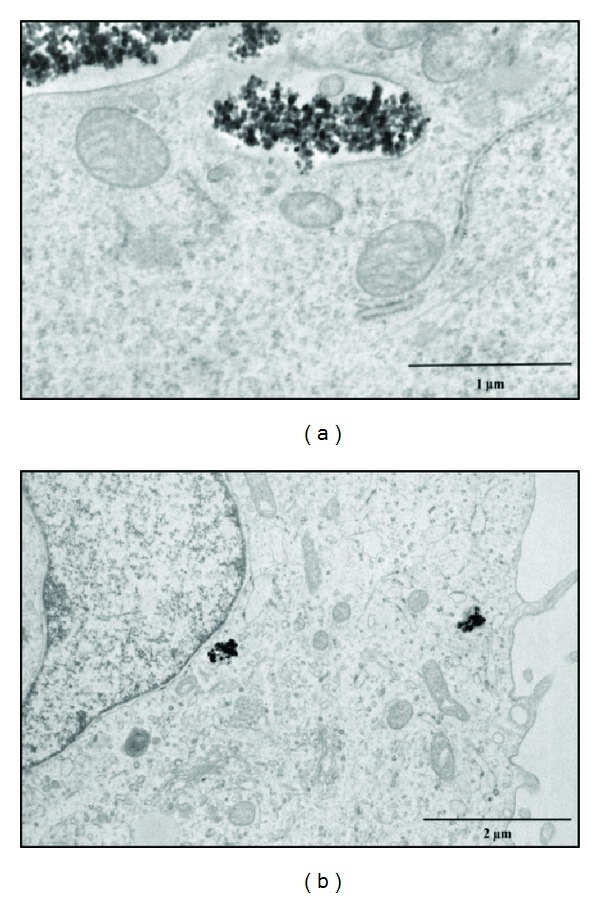
TEM pictures of SKOV-3 cells exposed for 30 min to Fe_3_O_4_@CMCS electrostatic (a) and covalent (b).

From our observations, the internalization did not seem to be influenced by the coating. Our hypothesis is confirmed by TEM picture (Figure 13) that did not show appreciable differences in cellular localization between Fe_3_O_4_@CMCS electrostatically or covalently bound and NP@heparin (Figures 11 and 12).

## 4. Discussion

In recent years, the use of NPs, particularly MNPs, has expanded into biomedical research. Due to their unique properties such as small size, large surface area, and high reactivity, they are particularly suitable for diagnosis and therapy [1, 26–29]. Often, NPs have to be covered with molecules to get a core@shell system capable to bind bioactive molecules, stable in physiological fluids and possibly not toxic to the body. Among the innumerable coating materials, polymers such as heparin, dextran, carboxydextran, chitosan, and polyethylene glycol are considered more advantageous to satisfy the above-mentioned characteristics [30–33].

In particular, the literature reports several applications of NPs covered with heparin, ranging from use as imaging agent to apoptosis-induced agent in cancer cell, as well as components of nanodevices [34–36]. Unfortunately, this wide number of publications does not include toxicity studies of the synthesized systems. In particular, the literature lacks data on the comparison between the toxicity of core and core@shell. To try to fill this gap, in our laboratory, we have studied the characteristics and behavior of Co_3_O_4_, Fe_3_O_4_, and NiO NPs covered with heparin.

From our experiments resulted that the coating had significantly increased the colloid stability and hydrophilic property of metal NPs. In fact, the systems NP@heparin did not agglomerated thanks to the presence of negatively charged groups around the metallic core. The experiments on cytotoxicity, performed on SKOV-3 cells, have shown that heparin itself was not toxic within the range of the examined concentrations (see Figure 5(a)). Furthermore, as expected, Fe_3_O_4_@heparin was the less toxic system, while NiO@heparin was the most toxic one. Contrary to what one would expect, NP@heparin had not been found less toxic compared to the naked NPs for all the examined metals (see Figures 6, 7, and 8). Depletion of ATP content, observed in these experiments, could be due to the massive internalization of NP@heparin by the cells, phenomenon substantiated by Prussian blue staining for iron detection. Nevertheless, at the concentrations used in these experiments, internalized Fe_3_O_4_@heparin did not arrest mitosis process and nanoparticles were shared between the daughter cells. Further analysis by TEM have demonstrated that NP@heparin were already present inside the cell after 30 min of exposure (Figures 11(a), 11(b), and 11(c)). In this work, we have not investigated the mechanisms of internalization even though, as shown in Figures 11(a), 11(b), and 11(c) and as reported by the literature [37–39], endocytosis is certainly a possible way. Notwithstanding in our previous work we had observed the presence of NPs also in the mitochondria and in the nuclei [40], in these experiments NPs were confined only in cytoplasmic vesicles, even though, sometimes, the vesicle size was so enormous to modify nuclear shape and/or cause mechanical damages to the cell (see Figure 12). When the endocitotic vesicles had sizes that did not justify the mechanical damage, we could assume that cell toxicity could be due to the release of metal ions by the NP system; this hypothesis was supported by the data of cell viability in which Fe_3_O_4_ NPs resulted the least toxic metal.

Chitosan, but even better CMCS, preferred because the carboxymethylation increases the chitosan solubility in physiological fluids, is widely studied for theranostic applications [41, 42]. Despite the Prussian blue staining indicated that Fe_3_O_4_@chitosan uptake was less efficient compared to that of Fe_3_O_4_@heparin, TEM analysis showed that no differences were noticeable between the two NP systems. Furthermore, as previously reported for heparin, the presence of negative charges on NP surface enhances interactions with the cell membrane facilitating cellular uptake [6, 38]. Thanks to its biocompatibility and the presence of active functional groups (amino, carboxyl, and hydroxyl), CMCS is a valid instrument to design novel biocompatible materials with tailored chemical and biophysical properties [43–46]. Despite the wide use of CMCS little or nothing is known about its behavior when it is associated with metal NPs. This lack of data suggested us to evaluate the properties and the potential toxicity of the system Fe_3_O_4_@CMCS itself and compared to the naked Fe_3_O_4_NPs. For our studies, we have set up two different systems: Fe_3_O_4_@CMCS-electrostatic bound and Fe_3_O_4_@CMCS-covalent bound. The interest in coating Fe_3_O_4_NPs by covalent bond resided in an attempt to get a system characterized by a more stable shell in hydrophilic fluids. 

Our studies on cell viability confirmed the biocompatibility of free CMCS at the tested conditions. When cells are exposed to Fe_3_O_4_@CMCS electrostatic, viability decreases with the same trend of Fe_3_O_4_NPs treatment (Figures 9(a) and 9(b)). The higher toxicity observed for Fe_3_O_4_@CMCS-covalent bond (Figure 9(c)) suggested that the method of preparation of the NPs could influence the cellular response.

Also in this case, uptake by SKOV-3 cells was relevant showing massive internalization already after 30 min with NPs stored in cytoplasmic vesicles (Figure 13) with no detectable difference between NP@heparin and Fe_3_O_4_@CMCS.

Our results have confirmed the data present in the literature about the biocompatibility of heparin and CMCS and their capability to get stable suspensions in hydrophilic fluids when conjugated to metal NPs, but not the ability to reduce the cytotoxicity of metal NPs coated with these polymers. Nevertheless, it is difficult to compare data derived from different experimental conditions such as different concentration ranges rather than diverse cell types which can give diverse responses to the same treatment [2, 47]. Moreover, the published data are often related to the whole system prepared and not to the single component, as we did, then the comparison is very difficult if not impossible.

In conclusion, the reactive groups, present on the surface of core@shell systems that we have synthesized, provide the opportunity for further functionalization so that a variety of biomolecules may be immobilized to enhance specific cell recognition for their use in targeting studies. Moreover, as regards Fe_3_O_4_NPs, even though the coating does not reduce their toxicity, the amount of NPs present in the systems is usually so low to render their toxicity negligible. Furthermore, due to their magnetic properties, Fe_3_O_4_NPs can be directed to the site of interest thanks to an external magnet. From this point of view, they could be promising tools as drug carrier for diagnosis and therapy.

## Supplementary Material

We have evaluated by two way ANOVA the cytotoxicity data which are plotted in the text figures.. Differences in cytotoxicity due to exposure time resulted almost always significant. Those due to coating resulted definitely significant only for Co_3_O_4_ NPs vs Co_3_O_4_@heparinand and Fe_3_O_4_ NPs vs Fe3O4@CMCS.
