# Supplementary material for: Heparin and Carboxymethylchitosan Metal Nanoparticles: An Evaluation of Their Cytotoxicity
Source: Biomed Res Int. 2013 Feb 18;2013:314091. doi: 10.1155/2013/314091 (PMC3590581; doi:10.1155/2013/314091)
Supplement: Supplementary file 1 — We have evaluated by two way ANOVA the cytotoxicity data which are plotted in the text figures.. Differences in cytotoxicity due to exposure time resulted almost always significant. Those due to coating resulted definitely significant only for Co3O4 NPs vs Co3O4@heparinand and Fe3O4 NPs vs Fe3O4@CMCS. [file 314091.f1.pdf]

Supplementary material

We have evaluated by two way ANOVA the cytotoxicity data which are plotted in the text figures. Differences in cytotoxicity due to exposure time resulted almost always significant. Those due to coating resulted definitely significant only for Co_3O_4 NPs vs Co_3O_4@heparinand and Fe_3O_4 NPs vs Fe_3O_4@CMCS.

* p≤0.05; ** p≤0.01; *** p≤0.005.

Co_3O_4 NPs	Between Co_3O_4 NPs/Co_3O_4@heparin		Among times	
5µg	0,00203	***	< .0001	***
10µg	0,00189	***	< .0001	***
20µg	0,00747	**	< .0001	***
40µg	0,00561	**	< .0001	***
60µg	0,00608	**	< .0001	***
100µg	0,00462	***	< .0001	***

Table 1: Two Way ANOVA on Co_3O_4 NPs and Co_3O_4@heparin.

Fe_3O_4 NPs	Between Fe_3O_4 NPs/Fe_3O_4@heparin		Among times	
5µg	0,34495		0,07401	
10µg	0,20480		0,00119	***
20µg	0,03442	*	< .0001	***
40µg	0,01517	*	0,00039	***
60µg	0,02287	*	0,01982	*
100µg	0,05681		0,08248	

Table 2: Two Way ANOVA on Fe_3O_4 NPs and Fe_3O_4@heparin.

NiO NPs	Between NiO NPs/NiO@heparin		Among times	
5µg	0,06262		< .0001	***
10µg	0,15043		< .0001	***
20µg	0,00841	**	< .0001	***
40µg	0,02880	*	< .0001	***
60µg	0,26751		< .0001	***
100µg	0,54143		< .0001	***

Table 3: Two Way ANOVA on NiO NPs and NiO@heparin.

Fe_3O_4 NPs	Between Fe_3O_4 NPs/Fe_3O_4@CMCS		Among times	
5µg	< .0001	***	0,00022	***
10µg	< .0001	***	0,00024	***
20µg	< .0001	***	< .0001	***
40µg	< .0001	***	< .0001	***
80µg	< .0001	***	< .0001	***
160µg	< .0001	***	< .0001	***

Table 4: Two Way ANOVA on Fe_3O_4 NPs and Fe_3O_4@CMCS- covalent.
